# Exploring secondary optical transitions: a study utilizing the DITM method, and enhanced photocatalytic properties in Ni-doped CuSe

**DOI:** 10.1038/s41598-024-58528-3

**Published:** 2024-04-02

**Authors:** Nader Ghobadi, Mohammad-Reza Zamani Meymian, Milad Fallah

**Affiliations:** 1 Department of Physics, Faculty of Science, Malayer University, Malayer, Iran; 2 Department of Solid-State Physics, Faculty of Physics, Iran University of Science & Technology, Tehran, Iran

**Keywords:** Electrical and electronic engineering, Photocatalysis, Engineering, Materials science, Physics

## Abstract

This study explores the simultaneous presence of two metal ions of Nickel (Ni) and Copper (Cu) on the formation of a metal selenide (Ni-doped CuSe) in an alkaline environment. The impact of Ni ions on creating the second optical transitions is investigated. Different concentrations amounts of Ni ions (0.01, 0.02, and 0.03 mol) are utilized to produce Ni-doped CuSe semiconductor thin films through a chemical solution deposition method with deposition times varying from 3 to 6 h. Absorbance spectra are employed to determine the band-gap, while Field Emission Scanning Electron Microscopy is utilized for morphological analysis. Structural and elemental analyses are conducted using X-ray Diffraction and Energy Dispersive X-ray Spectroscopy techniques. Additionally, a relatively innovative approach for determining the optical transitions, termed the Derivation Ineffective Thickness Method (DITM), is employed. DITM eliminates the need for thin film thickness and assumptions about the type of transition (direct or indirect) for band-gap calculation. Moreover, a comparison is made between the band-gap obtained from the Tauc model and the transitions obtained by DITM method. Furthermore, it is demonstrated that the optical transitions exhibit two distinct band-gaps associated with nickel selenide (NiSe) as second transition and copper selenide (CuSe) as fundamental transition. The presence of Ni is also found to enhance crystal quality. The study also briefly explores the improved photocatalytic properties of CuSe in the presence of Ni.

## Introduction

In semiconductors, the importance of doping is widely acknowledged for its impact on electrical and optical band-gaps, electron transport, and other electro-optical properties. Doping is the process of adding impurities to a semiconductor to modify its electrical properties. This crucial technique is employed to control the conductivity of the semiconductor and alter the number of charge carriers. When dopant atoms substitute semiconductor atoms in the lattice, an electron–hole pair is generated as a result of electronic states changes and transfers. The effects of doping extend beyond mere conductivity control. They include an increase in electron and hole mobility and a decrease in the threshold voltage, making devices more responsive and enhancing their electrical, optical and photocatalytic properties. Among these parameters, the control of the band-gap holds special significance because of its effects on electrical and optical characteristics. Although band-gap engineering has always attracted interest across all semiconductors, thin film semiconductors have intensified the attention due to a low surface-to-volume ratio. The key factor in band-gap manipulation lies in altering energy levels. In this context, transition metal ions, when employed as dopants, are well-recognized for their capability in inducing new mid-gap states, consequently adjusting Fermi levels, generating colored emissions, and influencing primary carrier recombination processes. Metal doping, particularly for mid-gap state creation, plays a foundational role in band-gap modulation, thereby enhancing the performance of thin films in applications ranging from LEDs, photocatalysts, to lasers^1–6^. Furthermore, metal doping and subsequent band-gap engineering appears to have a fundamental influence in quantum dots and semiconductor nanoparticles, particularly when presented in the form of thin films. This promises advanced electronic and optoelectronic devices with unique properties resulting from the quantum confinement effect^7,8^.

Selenides, as materials actively involved in the thin film industry, represent a category of chemical compounds wherein controlling the band-gap leads to more advanced applications. Among them, metal selenides hold particular importance on account of their diverse applications in energy storage, chemical catalysis, gas-sensing systems, and medicine. The fabrication of metal selenide thin films by two different metal ions (through metal-doping of metal selenides) appears to yield functional outcomes, especially concerning structural aspects and achieving a desired band-gap. In this regard, copper selenide (CuSe), commonly used in these areas, can improve its structure and band-gap by incorporating other metals as dopants, such as nickel (Ni), copper (Cu), and lead (Pb), leading to new band-to-band optical transitions^3,9–13^. In existing literature, there is no specific instance of research on nickel-doped copper selenide (Ni-doped CuSe) thin films. However, Patal et al.^11^ focused on Ni-doped ZnCuSe nanoparticles with a focus on antimicrobial properties. Moreover, Wang et al.^13^ produced Ni-doped CuSe nanowires, exploring their tunable optical and magnetic properties. Therefore, it appears that this research (Ni-doped CuSe thin film) presents a relatively less-studied area.

Beyond experimental research, the Tauc model is a well-established approach for calculating the optical band-gap in semiconductors^14,15^. In Tauc model formula, it is essential to determine the type of transition as direct or indirect, which governs n (transition index). Considering CuSe with a direct transition, n = 1/2 is used for it^16^. However, materials may introduce natural disorders into the optical band-gap transition procedure. To mitigate these irregularities, a realistic method like the Derivation of Ineffective Thickness Method (DITM) seems to be necessary. This method enables the determination of band-gap energy without presupposing the type of transition, offering a more precise estimation of the optical band-gap and transition nature. Ghobadi^17,18^, who introduced this method, demonstrated that the DITM can serve as an effective alternative to the Tauc model for determining the band-gap. The advantage of DITM lies in its reliance on absorbance measurement, eliminating the need for coefficient absorption (α) and the film thickness. On the other hand, he concluded that the DITM provides more precise results of optical band-gap and the nature of optical transitions in materials such as cadmium selenide (CdSe) and aluminum-doped zinc oxide (AZO) compared to both the Tauc model and the ineffective thickness method (ITM). Rezaee^19^ conducted a comparison between DITM and ITM in Ag–Cu–Pd alloy thin films, revealing good agreement between the results. However, she indicated that the advantage of the DITM method is its ability to determine the optical transition type without any presuppositions. Mehrparvar^20^ proved that the DITM method allows for a more realistic calculation of the transition index in cobalt selenide (CoSe). Similarly, Shiravand^21^ obtained analogous results in the context of AZO and the DITM method. Ghobadi^22^ worked on CoSe and asserted that DITM outperforms the Tauc plot in deriving the energy band-gap and determining the transition index. This superiority is attributed to DITM's ability to eliminate extrapolation uncertainties and the lack of need for transition index presumptions.

While various methods exist for thin film deposition, chemical approaches are specifically interesting due to their capacity for precise control over dopant concentration quantity, layer uniformity, and cost-effectiveness. In chemical methods, it becomes feasible to manipulate the concentration number of atoms in the layers at different levels, a flexibility which is not always possible with other methods. As an instance, traditional methods like sputtering pose challenges in achieving uniform impurity distribution among particles. The approach of fabricating metal selenide thin films using two different metals at different concentrations amounts is a new experience that yields compelling physical outcomes. CuSe, synthesized through a chemical method, exhibits a large energy gap under specific alkaline conditions. Introducing another metal ion during deposition can result in creation of new physical properties, enhancement of layer quality, and lower energy band-gap^23–30^. In this research, a Ni-doped CuSe thin film is produced via the chemical solution deposition method at different deposition times (3, 4, 5, and 6 h) with varying Ni concentrations amounts (0.01, 0.02, and 0.03 mol) as dopant. Subsequently, the fundamental and second optical band-gap, assessed through the DITM method, is compared with the results obtained from the Tauc model. Finally, the effect of Ni on the photocatalytic activity of CuSe is presented.

## Experimental procedures

To fabricate Ni-doped CuSe (NiCuSe) thin films through the chemical solution deposition process, the copper nitrate trihydrate (Cu(No_3_)_2_ − 3H_2_O), nickel nitrate hexahydrate (NiN_2_O_6_ − 6H_2_O), 25% ammonia (NH_3_), and selenium powder (Se) in their pure form (> 99% pure, Merck Co., Darmstadt, Germany) is used. Using the relation of C_M_ = 1000(cc) × (m(gr)/V(cc)) × M(gr) in which the C_M_ is molarity, m is the desired weight, V is the desired volume, and M is the molar mass, the desired quantity of selenium powder is mixed with sodium sulfite in a tenfold concentration to create a solution. The molarity of Se, Cu and Ni are indicated in Table 1. Subsequently, this solution undergoes stirring at 80 degrees Celsius for 12 h, constituting the initial selenium or non-metal ions. and to prepare a 100 mL solution of sodium selenosulfate (Na_2_SeSO_3_) with a molarity of 0.07 as the selenium source, the required amount of sodium sulfide (Na_2_SO_3_) was dissolved in distilled water under magnetic stirring at 80 degrees Celsius. After complete dissolution, the necessary amount of Se powder was added to the solution, and stirring continued at the same temperature for 12 h. This solution provides us with the selenium ions (Se^2−^) or initial non-metallic ions source.

$$ {\text{Na}}_{{2}} {\text{SO}}_{{3}} + {\text{H}}_{{2}} {\text{O}} \to {\text{2Na}}^{ + } \left( {{\text{OH}}} \right) + {\text{H}}_{{2}} {\text{O}} + {\text{SO}}_{{3}}^{{{2} - }} $$

$$ {\text{2Na}}^{ + } + {\text{So}}_{{3}}^{{{2} - }} + {\text{Se}} + {\text{H}}_{{2}} {\text{O}} \leftrightarrow {\mathbf{Na}}_{{\mathbf{2}}} {\mathbf{SeSo}}_{{\mathbf{3}}} + {\text{H}}_{{2}} {\text{O}} $$

$$ {\text{Na}}_{2} {\text{SeSO}}_{3}  + {\text{OH}}^{ - }  \leftrightarrow {\text{Na}}_{2} {\text{SO}}_{4}  + {\text{HSe}}^{ - }  $$

$$ {\text{HSe}}^{ - } + {\text{OH}}^{ - } \leftrightarrow {\text{H}}_{{2}} {\text{O}} + {\mathbf{Se}}^{{{\mathbf{2}} - }} $$

**Table 1:** Fundamental and second optical band-gaps of CuSe and Ni-doped CuSe samples prepared with different amount of Ni (0.01, 0.02, and 0.03 mol) under varying deposition time (3, 4, 5, and 6 h) by chemical deposition method through Tauc's plot as well as DITM method.

Sample No.	CuSe	Ni-doped CuSe											
	S13	S1	S2	S3	S4	S5	S6	S7	S8	S9	S10	S11	S12
Content on Se (mol)	0.07	0.07	0.07	0.07	0.07	0.07	0.07	0.07	0.07	0.07	0.07	0.07	0.07
Content on Cu (mol)	0.07	0.07	0.07	0.07	0.07	0.07	0.07	0.07	0.07	0.07	0.07	0.07	0.07
Content on Ni (mol)	–	0.01	0.01	0.01	0.01	0.02	0.02	0.02	0.02	0.03	0.03	0.03	0.03
Deposition Time (hours)	5	3	4	5	6	3	4	5	6	3	4	5	6
*Tauc model*													
Fundamental band gap, CuSe (eV)	3.99	–	3.77	–	3.62	3.56	3.47	3.40	3.24	3.74	3.70	3.50	3.38
Second band gap, Ni-doped CuSe (eV)	–	–	2.07	–	1.97	1.72	1.70	–	1.52	–	–	–	1.37
*DITM Method*													
Fundamental band gap, CuSe (eV)	–	4.1	4	4.1	3.93	4.02	3.96	4.02	4.02	4.05	4.04	4.04	4.02
Second band gap, Ni-doped CuSe (eV)	–	–	2.13	2.2	2.28	1.75	1.65	1.50	1.53	1.55	1.49	1.41	1.4

The subsequent step involves generating a metal solution, comprising specified amounts of Cu(NO_3_)_2_ − 3H_2_O and NiN_2_O_6_ − 6H_2_O. To form a Copper nitrate (Cu(NH_3_)_4_^2+^), NH_3_ is added to the solution resulting from the dissolution of Cu(NO_3_)_2_ − 3H_2_O in distilled water. Subsequently, by adding an additional amount of NH_3_, we proceed to the Cu(NH_3_)_4_^2+^ according to the reactions outlined below. The purpose of adding NH_3_ is to ionize the Cu^2+^ ions from the solution.

$$ {\text{Cu}}\left( {{\text{NO}}_{{3}} } \right)_{{2}} - {\text{3H}}_{{2}} {\text{O}} + {\text{H}}_{{2}} {\text{O}} \leftrightarrow {\text{Cu}}\left( {{\text{OH}}} \right)_{{2}} + {2}\left( {{\text{NO}}\left( {{\text{OH}}} \right)_{{3}} } \right) $$

$$ {\text{Cu}}\left( {{\text{OH}}} \right)_{{2}} + {2}\left( {{\text{NO}}\left( {{\text{OH}}} \right)_{{3}} } \right) + {\text{NH}}_{{3}} \leftrightarrow {\text{Cu}}\left( {{\text{OH}}} \right)_{{2}} + {2}\left( {{\text{NH}}_{{4}} } \right){\text{NO}}_{{7}} {\text{OH}} $$

$$ {\text{Cu}}\left( {{\text{OH}}} \right)_{{2}} + {2}\left( {{\text{NH}}_{{4}} } \right){\text{NO}}_{{7}} {\text{OH}} + {\text{4NH}}_{{3}} \leftrightarrow {\mathbf{Cu}}\left( {{\mathbf{NH}}_{{\mathbf{3}}} } \right)_{{\mathbf{4}}}^{{{\mathbf{2}} + }} + {2}\left( {{\text{NH}}_{{4}} } \right){\text{NO}}_{{7}} \left( {{\text{OH}}} \right)_{{3}} $$

$$ {\text{Cu}}\left( {{\text{NH}}_{{3}} } \right)_{{4}}^{{{2} + }} \leftrightarrow {\text{4NH}}_{{3}} + {\mathbf{Cu}}^{{{\mathbf{2}} + }} $$

To form a metal complex, NH_3_ is added to the metal nitrate solution. This complex prevents abrupt deposition upon the mixture of metallic and non-metallic solutions, allowing controlled deposition. To introduce Ni impurities, a solution containing Ni (using NiN_2_O_6_ − 6H_2_O) similar to the preparation of copper nitrate solution is utilized. And before adding NH_3_ to the copper nitrate solution, the nickel-containing solution is mixed with the copper nitrate solution. The primary reason for formation of metal selenides instead of metal oxides, in the presence of NH_4_OH when using selenium powder can be related to the reaction between selenium ions and metal ions in the presence of NH_4_OH. Metal oxides often exhibit instability in alkaline environments due to their oxidation state properties, and they might transform into metal selenides. One of the possible reactions leading to the formation of metal selenides is as follows: 2Se^2−^ + M^n+^ + 2OH^−^⟷MS + H_2_O_2_. In this reaction, selenium ions (Se^2−^) combine with metal ions (M^n+^) and hydroxide ions (OH^−^), resulting in the formation of metal selenide (MS) and hydrogen peroxide (H_2_O_2_).

For washing the substrates, an ultrasonic treatment in water and alcohol solutions is used. The substrates are positioned at a 20-degree angle to the container wall, and in the final step, the solution is poured into the container with temperature control via a heater. The crystal structure of the thin films is analyzed using a Philip X-ray Diffractometer (XRD) with CuKα (α = 1.5425 Å) radiation. Optical properties are examined through UV–Vis spectrometry (Perkin-Elmer, UV/VIS Spectrometer Lambda 25-USA) within the wavelength range of 190–1100. Film microstructure is observed using a Field Emission Scanning Electron Microscope (FESEM) employing the Tescan Mira (III) FESEM instrument, equipped with Energy Dispersive X-ray spectrometer (EDS) for elemental analysis. Film elemental composition analysis was conducted utilizing X-ray Photoelectron Spectroscopy (XPS) (Bestec, Berlin, Germany) in which the instrument utilized a monochromatic Mg Kα radiation (1253.6 eV) X-ray source for the analysis.

## Results and discussion

### Structural properties

**Figure 1:**
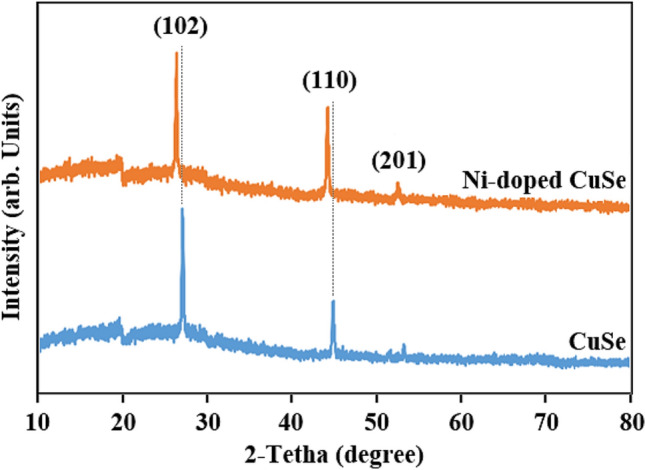
XRD patterns for CuSe and Ni-doped CuSe thin films after 6 h of deposition and 0.03 mol of Ni.

In Fig. 1, the XRD patterns of CuSe and Ni-doped CuSe with 0.03 mol of Ni after 6 h of deposition validate the hexagonal phase of CuSe and Ni-doped CuSe, consistent with hexagonal CuSe JCPDS card no. 00-006-0427^31,32^. According to Fig. 1, the peaks associated with the (102), (110), and (201) planes are clearly identifiable, indicating the CuSe. The intensity of these peaks increases after the addition of Ni. The inclusion of Ni as an impurity within the crystal lattice of CuSe does not perturb the hexagonal phase of CuSe or the (102) plane as a preferred orientation. This indicates that the Ni is well inserted into the crystal structure of CuSe, and no alterations are observed in this structure after incorporation of Ni. Furthermore, owing to the slightly larger ionic radius of Ni compared to Cu, a lower-theta shift is observed in the Ni-doped CuSe XRD peaks (dashed line) compared to the CuSe peaks, confirming the effective penetration of Ni into the CuSe lattice structure with larger particle size. The sharper peak in Ni-doped CuSe, in comparison to CuSe, suggests that the presence of Ni contributes to a higher crystalline quality of CuSe due to higher degree of order, crystallization and arrangement by filling the vacancies in the lattice network^33–38^. Additionally, the larger ionic radius of Ni, leading to increased spacing between crystal planes during the replacement of Cu with Ni, is another factor contributing to a larger crystalline size after doping^39–41^. In this regard, by calculating the Scherrer’s equation (D = Kλ/βCosθ) in which D represents the crystallite size, K is a constant known as the shape factor (equal to 0.9), λ is the wavelength of X-ray radiation (1.54 Å), β is the Full Width at Half Maximum (FWHM) based on 2θ in radians, and θ in cosθ is related to the XRD peak position as Bragg's diffraction angle (θ = 2θ/2 in radians), the crystallite sizes in the CuSe and 0.3 mol Ni–CuSe samples after 6 h of annealing (Fig. 1) have been determined as 24.44 and 26.75 nm, respectively. Moreover, the averae crystallite sizes are 20.46 and 22.06 nm for CuSe and NiCuSe, respectively. The increase in crystallite size, indicative of an improvement in crystal quality in NiCuSe compared to CuSe, is consistent with visual observations in Fig. 1^42^. However, the observed lower-theta shift and increased grain crystalline size after Ni incorporation imply that at higher Ni concentrations amounts or under extended deposition times, there is a potential risk of disrupting the crystal network due to elevated strain, dislocation density, and mismatches between the host cation (Cu^2+^) and the dopant cation (Ni^2+^)^11^.

**Figure 2:**
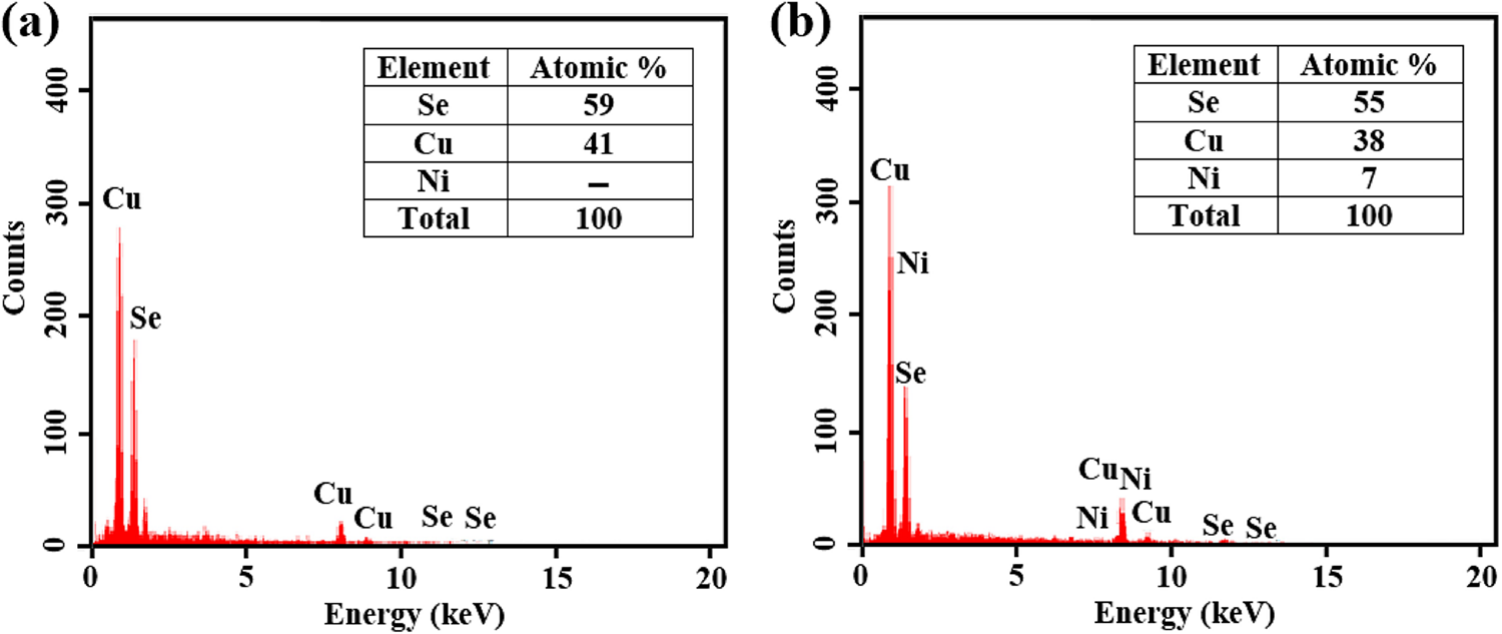
EDS pattern of (**a**) CuSe thin film after 6 h of deposition and (**b**) Ni-doped CuSe thin film after 6 h of deposition and 0.03 mol of Ni.

Chemical analysis was performed using EDS for both CuSe and Ni-doped CuSe thin films deposited on a glass substrate. Figure 2a and b depict the EDS patterns for CuSe and Ni-doped CuSe thin films, respectively. As shown in Fig. 2, the average atomic percentage of Cu:Se for the sample with 0.03 mol of Ni and a deposition time of 6 h is 41%:59%. Furthermore, the average atomic percentage of Cu:Ni:Se is reported as 38%:7%:55%.

**Figure 3:**
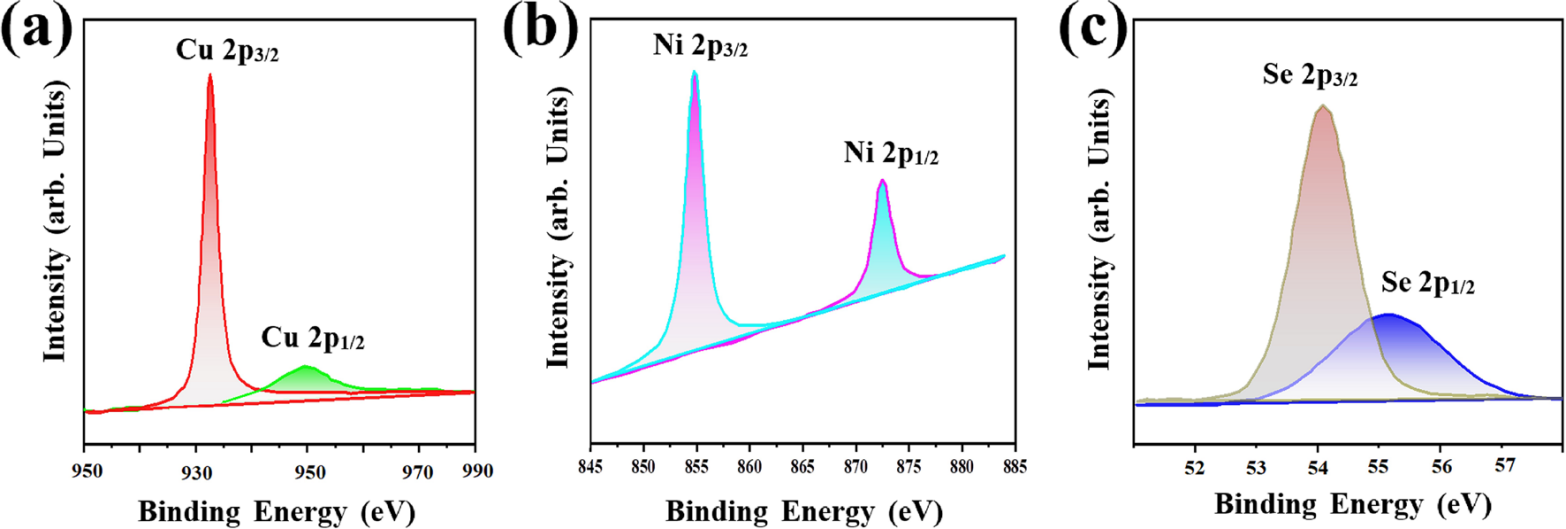
XPS spectra for core level peak regions of (**a**) Cu 2p, (**b**) Ni 2p, and (**c**) Se 2p.

NiCuSe particles were examined using X-ray Photoelectron Spectroscopy (XPS) to understand the composition and bonding of Ni, Cu, and Se. In Fig. 3a–c, detailed XPS spectra for Ni 2p, Cu 2p, and Se 2p core levels reveal binding energy peaks at 933.2 and 950 eV for Cu 2p3/2 and Cu 2p1/2, indicating the presence of Cu^2+^ ions in NiCuSe. Similarly, peaks at 54.1 and 55.2 eV for Se 2p3/2 and Se 2p1/2 suggest the involvement of Se^2−^ in NiCuSe. Furthermore, the peaks at 854.7 and 872.3 eV are attributed to the Ni 2p3/2 and Ni 2p1/2 components, respectively, suggesting Ni^2+^, all aligning with reported values in existing literature^31,43–47^.

### Surface morphology

**Figure 4:**
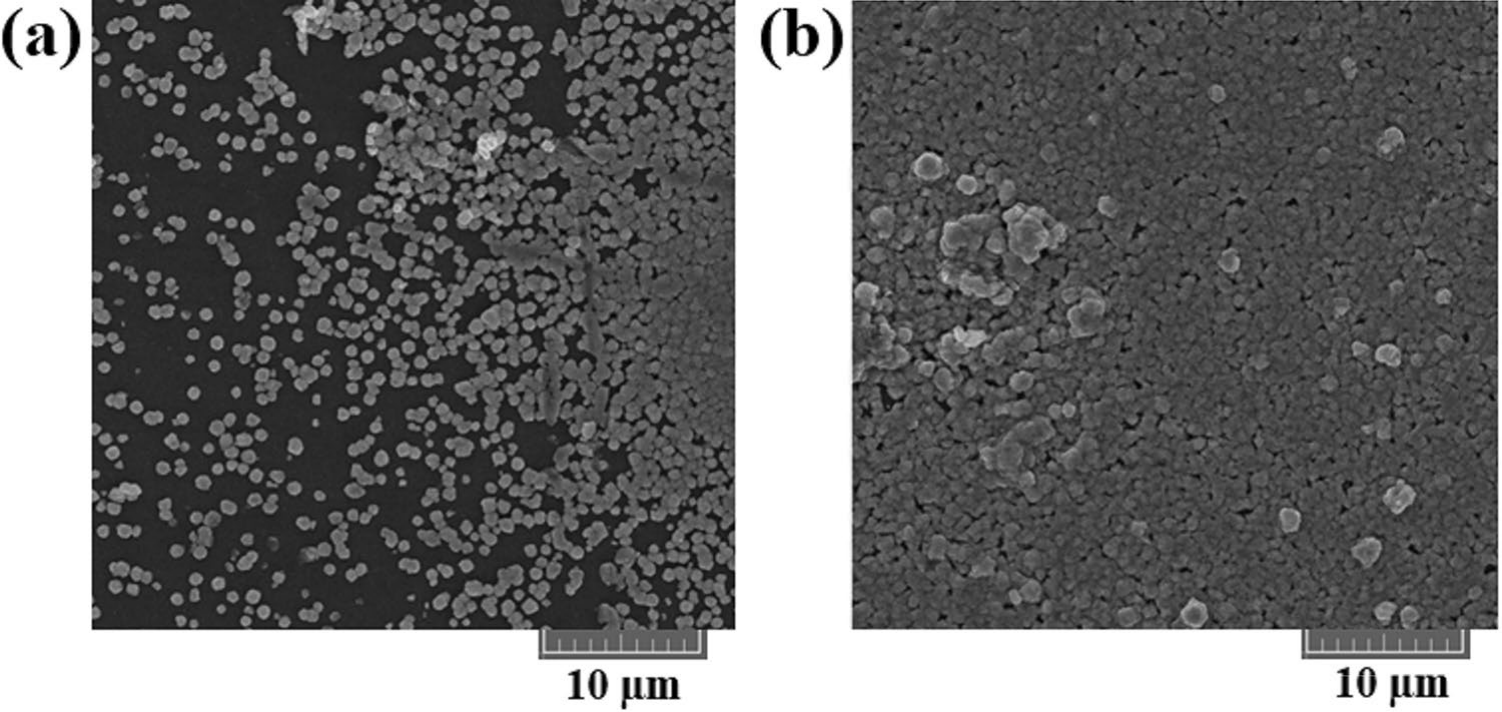
SEM images of (**a**) CuSe thin film after 6 h of deposition and (**b**) Ni-doped CuSe thin film after 6 h of deposition and 0.03 mol of Ni.

SEM micrographs in Fig. 4a and b reveal the morphology of pure CuSe and Ni-doped CuSe films, respectively. Notably, the Ni-doped CuSe exhibits densely packed particles, contrasting with the dispersed arrangement in pure CuSe. The introduction of Ni to CuSe structure results in a more compact structure, enhancing the coverage rate of film surface and connectivity of particles. Consequently, the addition of Ni contributes to the reduction of pinholes and induces some agglomeration. The Ni-induced agglomeration, while promoting uneven grain growth^48^, significantly diminishes surface discontinuity. This is attributed to the facilitated growth of Ni^2+^ ions, promoting nucleation and clusters formation^49,50^. Therefore, introducing Ni improves the overall quality of the substrate coverage and particles connectivity by decreasing the kinetic barrier of nucleus attachment rate, and by creating mid-gap defect states (based on Ni atoms as dopants) for clustering^51,52^. Furthermore, upon further analysis of SEM images, the average grain sizes in CuSe and NiCuSe are determined to be 0.94 and 1.03 µm, respectively, which is in consistent with the larger crystallite sizes (resulted by XRD data) in the sample with the presence of Ni.

#### Optical properties

**Figure 5:**
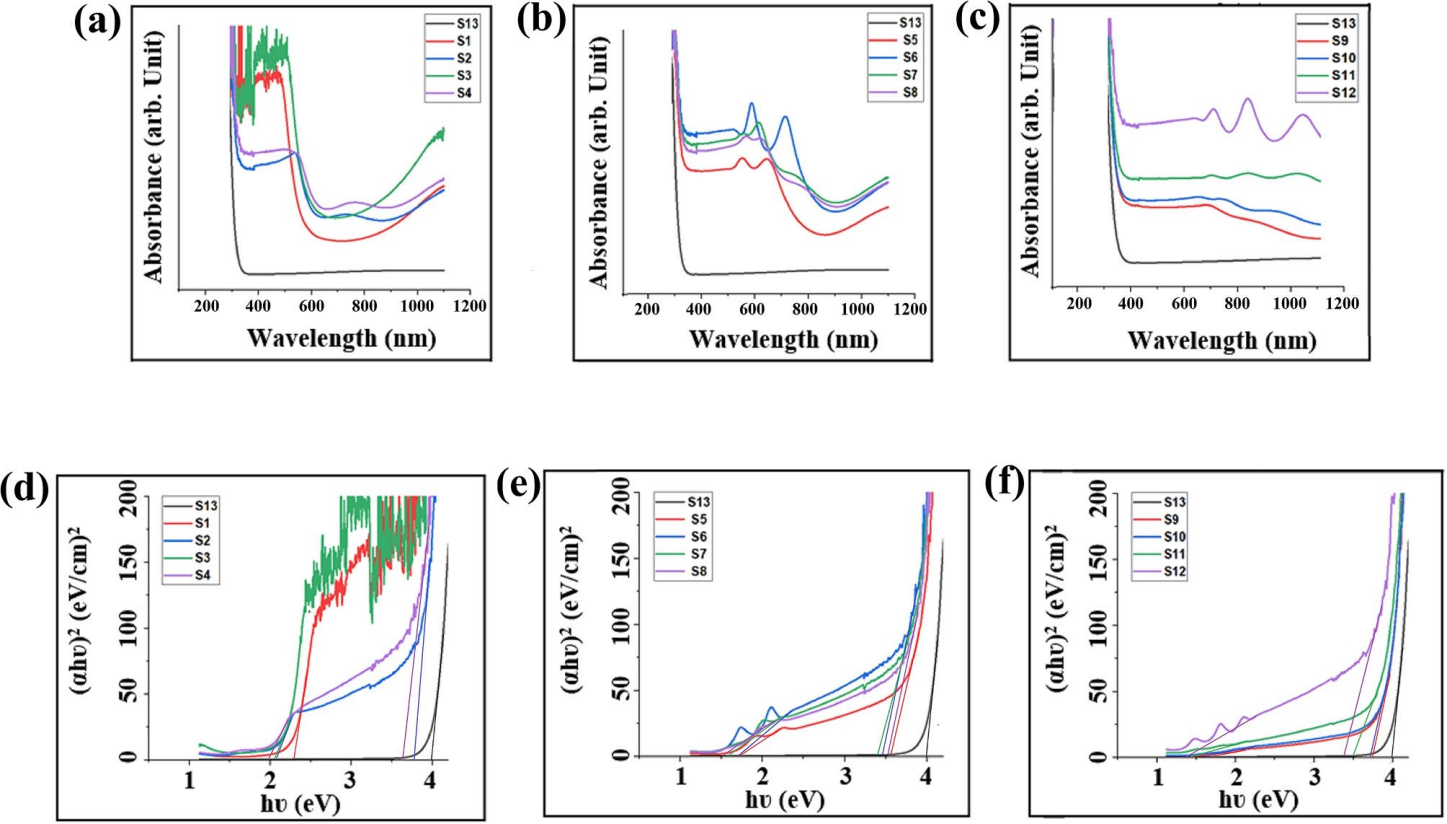
(**a**–**c**) Absorbance spectra of CuSe (S13) and Ni-doped CuSe (S1–12), and (**d**–**f**) band-gaps of CuSe (S13) and Ni-doped CuSe (S1–12) based on Tauc's plot.

To compare the band-gaps obtained from the Tauc model and the DITM method, the band-gap is initially determined using the Tauc model. In Fig. 5, Tauc's equation (αhν) = B(hν—Eg)^1/2^ is applied, where the direct band-gap of CuSe is determined by plotting (αhν)^2^ on the y-axis against hν on the x-axis. A tangent line is drawn on the curve where αhν = 0, and the point of intersection on the x-axis represents the optical band-gap^53^. The optical absorption spectrum (Fig. 5a–c) and the Tauc band-gaps (Fig. 5d–f) for thirteen samples are presented, including one for pure CuSe (S13) and twelve for Ni-doped CuSe samples (S1–S4 related to 0.01 mol of Ni, S5–S8 to 0.02 mol of Ni, and S9–S12 to 0.03 mol of Ni, each with varying deposition times ranging from 3 to 6 h, respectively). These samples were created at a deposition temperature of 50 degrees Celsius. Figures 5d, 4e, and f exhibit diagrams depicting multiple band-gap (including S2, S4, S5, S6, S7, S8, and S12), while in others, it is not clearly evident.

Given that no phase other than hexagonal is observed in the X-ray diffraction pattern of CuSe, the presence of two linear segments in the band-gap diagram cannot be attributed to the intrinsic defects or secondary phase of the material. Therefore, the first linear segment (first transition) at higher energy of Tauc plots is related to fundamental optical gap (energy gap between valence band [π-band] and conduction band [π *-band]). Moreover, the second linear segment (second transition) at lower energy of Tauc plots in the band-gap diagram is associated with the localized defects state originated from the dopant (energy gap between the HOMO [π-orbitals] and the LOMO [π*-orbitals]) ^54–56^. In other words, the presence of Ni and the NiSe bonds as well as various particle sizes are responsible for the multiple band-gaps observed in the material at lower energies. In more detail, particles of various sizes are produced, some of which are in the nanoscale dimensions and exhibit quantum confinement effects, leading to an inverse relationship between particle size and band gap. Moreover, these different particle sizes give rise to distinct boundaries that induce variations in the light absorption mechanism of the material. Therefore, the absorption edges in different samples are different, as confirmed in Fig. 5. These distinct absorption edges, appearing as step-like features, result in differences in the band gap. Additionally, Ni can infiltrate the CuSe lattice, occupying lattice vacancies and inducing its own band gap due to the bonds it forms. However, based on XRD and XPS results, it is established that the material fundamentally consists of CuSe. Nevertheless, with the presence of a small amount of Ni, secondary effects of Ni are observed without structural disruption^57,58^. Additionally, irregularities at the end of the spectra, regardless of being related to Ni, may be attributed to phonon oscillations or optical interactions. However, as can be seen in Table 1, the limitations of Tauc plots hinder the ability to obtain accurate band-gap values for all samples.

According to Table 1, as the molarity of Ni increases, the band-gap decreases in the cases of 0.01 and 0.02 mol of Ni. This is due to the presence of Ni energy states incorporated into the structure as well as reduction in vacancy-defect points of sub-lattice. In the presence of 0.01 mol of Ni, the fundamental band-gap (first transition) is distinctly discernible only for deposition times of 4 and 6 h as (S2) 3.77 and (S4) 3.62 eV, respectively. The fundamental optical band-gap value for the 0.02 mol Ni at deposition times of 3, 4, 5, and 6 h corresponds to (S5) 3.56, (S6) 3.47, (S7) 3.40, and (S8) 3.24 eV, respectively. However, in the case of 0.03 mol of Ni, an increase in the band-gap is observed. For 0.03 mol Ni, for deposition times of 3, 4, 5, and 6 h, the values are (S9) 3.74, (S10) 3.70, (S11) 3.50, and (S12) 3.38 eV, respectively. The value for (S13) pure CuSe is 3.99 eV. Considering the absence of unknown crystalline phases in the XRD, an increase in the fundamental band-gap range in the case of 0.03 mol of Ni compared to 0.02 mol, while this range is decreased in the case of 0.01 mol Ni to 0.02 mol, can be related to the formation of a degenerated semiconductor due to higher level of doping. Additionally, based on the data in the Tauc plots, it is observed that, at different molarities of Ni, an increase in the deposition time results in a reduction of the band-gap. This phenomenon is related to the improvement in nucleation conditions^59^ and higher kinetic energy of atoms. As depicted in Table 1, the visible second band-gap (second transition) values for samples S2, S4, S5, S6, S8, and S12 are 2.07, 1.97, 1.72, 1.70, 1.52, and 1.37, respectively.

Regarding the limitation of the Tauc plot, it should be noted that despite its widespread use in materials community, the extrapolation technique employed in the Tauc model is subjected to uncertainty originated from the background in Tauc plot. This can results in inaccurately obtained values of band-gaps. In this regard, one significant factor of inaccuracy arises from the surface imperfections like scratches, dust, grain boundaries, and crystal defects, introducing light scattering that manifests as absorption-like optical losses. Moreover In the case of defective or degenerate semiconductors, the observed optical absorption band-gap may deviate from the fundamental band-gap due to phenomena such as Burstein-Moss shifts or band-gap renormalization resulting from electron–electron and electron–ion interactions as well as the formation of dopant intra-band-gap states within the band-gap. So, in such instances, the direct application of the Tauc method leads to an inaccurate estimation of band-gap^60–63^.

#### Determination of transition type and derivation ineffective thickness method (DITM)

The DITM obviates the need for measuring the layer thickness and type of optical transition (direct or indirect). The entire process, utilizing the absorption spectra and derivation relationship, has been detailed in previous works and is succinctly outlined below.

Ln(A(υ)E) = mLn(K) + mLn(E − Eg). The derivative of Ln(A(υ)E) with respect to E(eV) is given by d{Ln[A(υ)E]}/d(E) = m/(E − Eg).

Discontinuity peaks in the d{Ln[A(υ)E]}/d(E) plot against E(eV) yield the precise value of the optical band-gap^17,21,64,65^.

**Figure 6:**
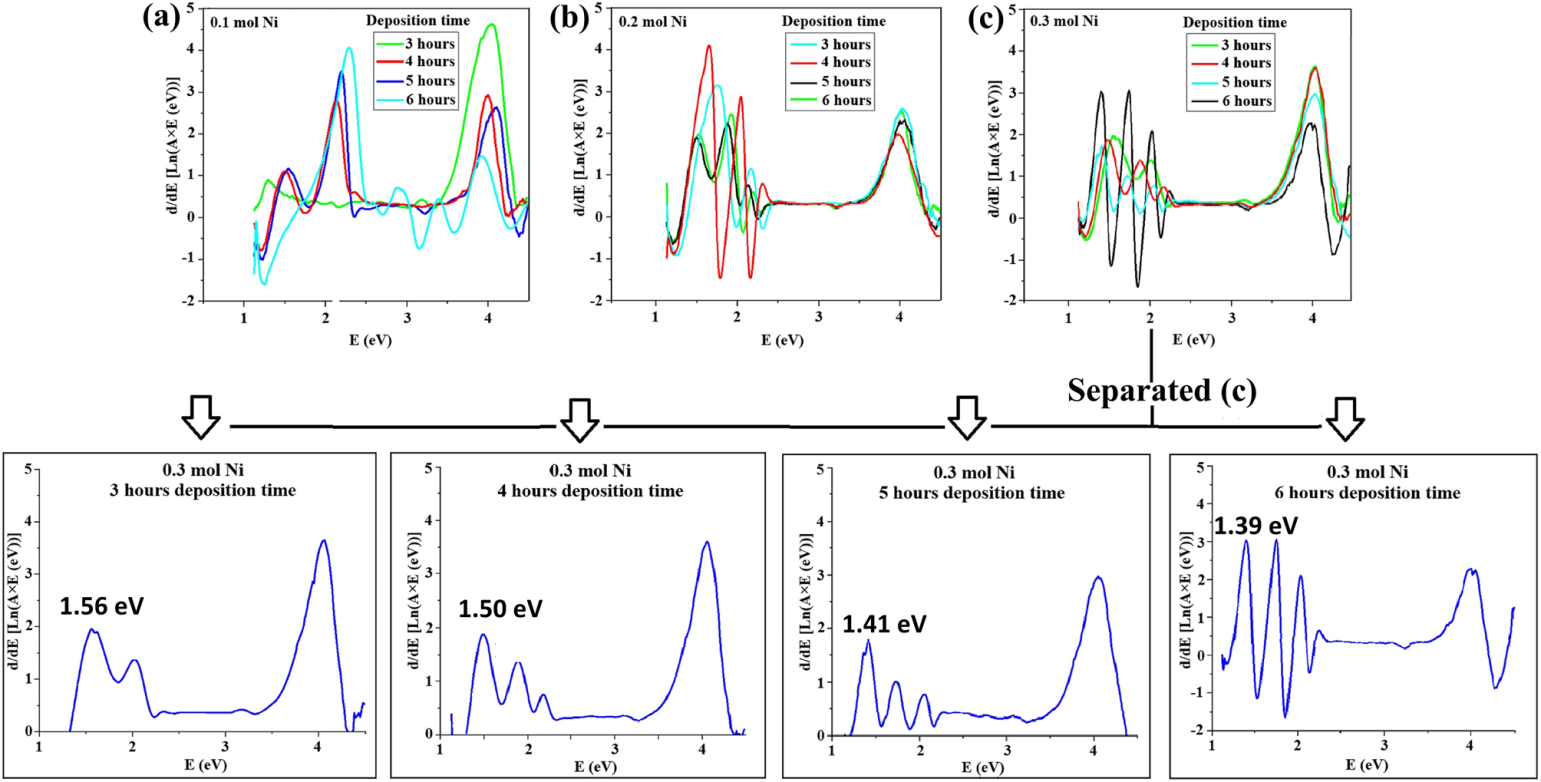
Diagram of d{Ln[A(υ)E]}/d(E) versus E(eV) for samples with different deposition time of 3, 4, 5, and 6 h and (**a**) 0.01 mol Ni, (**b**) 0.02 mol Ni, (**c**) 0.03 mol Ni as well as the separated diagram of (**c**).

Figure 6 illustrates plots of d{Ln[A(υ)E]}/d(E) versus E(eV) for Ni-doped CuSe samples with Ni amounts of 0.01 (Fig. 6a), 0.02 (Fig. 6b), and 0.03 (Fig. 6c) mol, and different deposition times (3, 4, 5, and 6 h), respectively. Alterations in deposition time can lead to changes in crystal structures, particle sizes, dislocation densities, and other factors, thereby affecting the electronic states within the material and consequently altering its electrical and optical properties^37,66^. Moreover, Fig. 6c presented in a separated manner for clarification. The introduction of Ni ions into CuSe is evidently observed to generate new optical transitions related to NiSe (second transition). This implies that even a small concentration amount of Ni ions results in new transitions in CuSe, thereby giving the multiple optical band-gaps. So, the presence of various energy band-gaps in Fig. 6 is attributed to the presence of Ni and its energy states, as well as the existence of different particle sizes following various deposition time as discussed earlier. The varying particle sizes can give rise to different energy band-gaps. Moreover, the possibility of forming NiSe particles separately, during the doping process, can lead to a different band-gap alongside Ni-doped CuSe particles. The values of the band-gaps in different samples are provided in Table 1. This presents a challenge between DITM and the Tauc model, making DITM more realistic.

In accordance with the results in Table 1 related to the band-gaps in the DITM section, the fundamental band-gap is positioned around 4 eV, similar to the Tauc model outcomes. However, unlike the Tauc model where half of the second transitions are not observed, here, the second transitions (related to Ni) are observed for all doped samples. Only in the sample with 0.01 mol of Ni, no second transition is observed, which is attributed to the insufficient formation of NiSe at this low Ni molarity with 3 h deposition time, which is the minimum condition. The band-gap values associated with the presence of Ni using the DITM model for 0.01 mol of Ni and deposition times of 4, 5, and 6 h are (S2) 2.13, (S3) 2.2, and (S4)2.28 eV, respectively. These values for the sample with 3, 4, 5, and 6 h of deposition and 0.02 mol of Ni are (S5) 1.75, (S6) 1.65, (S7) 1.5, and (S8) 1.53 eV, respectively. For samples with 0.03 mol of Ni, these values are (S9) 1.55, (S10) 1.49, (S11) 1.41, and (S12) 1.4 eV. The trend of increasing the fundamental band-gap range in the case of 0.03 mol Ni with the DITM model is observed similar to the results of the Tauc plot. Additionally, with the increase in Ni molarity and deposition time, the second band-gap related to Ni follows a decreasing path. This indicates that the presence of Ni has effectively contributed to the formation of NiSe bonds. The reduction of the band-gap which is attributed to the increase in the amount of Ni and its energy levels, as well as an extended deposition time which lead to a better and more nucleation process is another reason supporting the claim on formation of NiSe bonds. Furthermore, the presence of multiple peaks which are corresponds to the second band-gaps is related to NiSe bonds, potential molecular absorption of Ni, lattice distortions, and the different particle sizes that is formed. The criterion for selecting the main second band-gap in Table 1 is related to the peaks formed around the most intense peak in 3 h deposition (minimum condition for nucleation).

**Figure 7:**
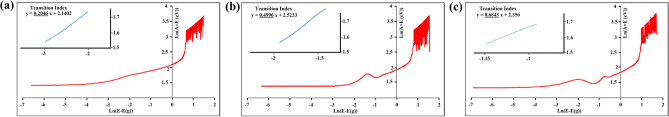
Diagram of Ln[A(υ)E] versus Ln (E − E(g)) for samples with 0.03 mol Ni and different deposition time of (**a**) 4, (**b**) 5, and (**c**) 6 h.

By plotting Ln[A(υ)E] against Ln (E − E(g)), it becomes possible to extract a transition index that characterizes the semiconductor nature optical transition. This transition index serves to discern whether the manufactured material possesses a direct or an indirect band-gap. The transition index values for Ni-doped CuSe, determined by the DITM method, are 0.2046, 0.4996, and 0.6845. These values indicate that the transitions in Ni-doped CuSe is direct, as they are close to 1/2 rather than 2. Figure 7 illustrates the aforementioned transition indexes.

### Photocatalytic activity

**Figure 8:**
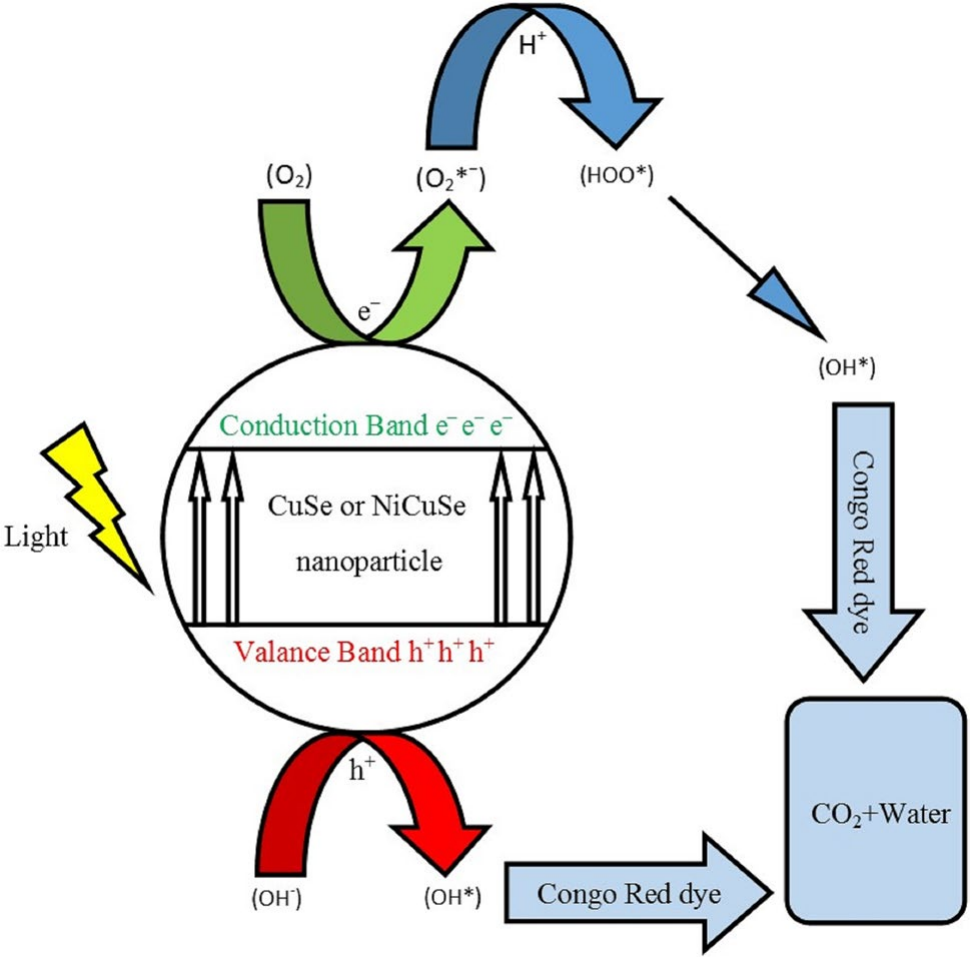
A typical illustrative diagram of photocatalytic activity.

**Figure 9:**
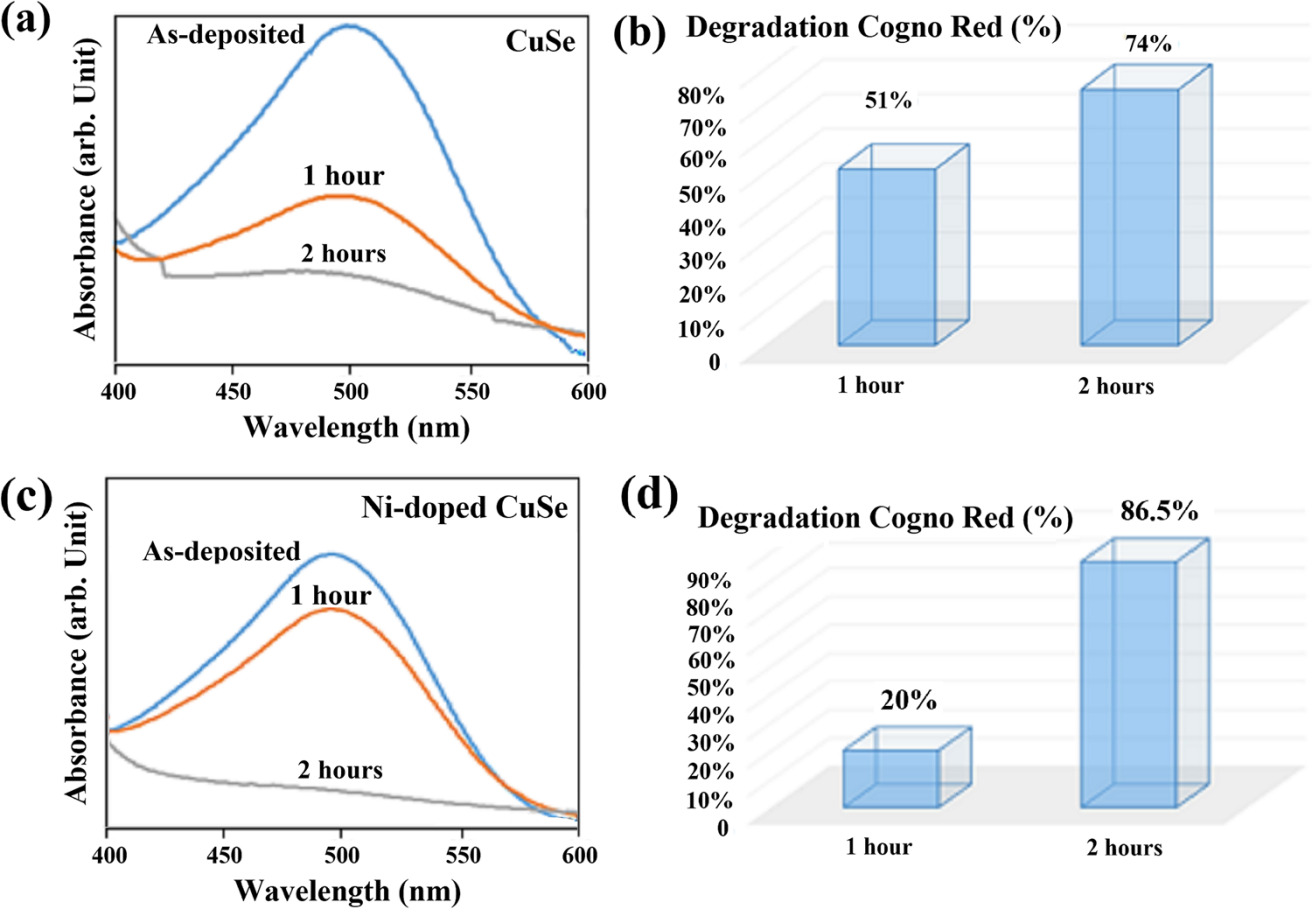
(**a**, **c**) Absorption spectral change of Congo Red over irradiation time in the presence of CuSe and Ni-doped CuSe thin films. (**b**–**d**) Degradation percentage of Congo Red following 1 and 2 h of irradiation with CuSe and Ni-doped CuSe thin films.

The photocatalytic characteristics of CuSe thin layers are assessed by quantifying the degradation rate of Congo Red (CR) solution, an environmentally impactful organic compound, under solar light. Here, the degradation of Congo Red solution was carried out in the presence of CuSe and NiCuSe photocatalytic layers. Initially, the layer was allowed to equilibrate in the dark for 30 min for adsorption/desorption balance, and then photocatalytic testing was performed for 1 and 2 h under UV light. The noticeable reduction in the absorbance peak of CR at a wavelength of 500 nm demonstrates the effective degradation capability of the CuSe and NiCuSe photocatalytic layers, as illustrated in Figs. 8 and 9. Thin films of CuSe, positioned on a glass plate, are employed to degrade CR solution (2 × 10^–6^ M) under solar irradiation. The degradation efficiency percentage of CR is calculated using the following relationship.

$$ {\text{Degradation }}\% \, = \, \left( {{\text{C}}0 \, - {\text{ C}}} \right) \, /{\text{ C}}0 \, \times { 1}00 \, = \, \left( {{\text{A}}0 \, - {\text{ A}}} \right) \, /{\text{ A}}0 \, \times { 1}00 $$

Here C0 stands for the initial concentration, and A0 signifies the initial absorption. Meanwhile, C and A represents the concentration and absorption, respectively, after the irradiation time throughout the process. Figure 8 schematically describes the photocatalytic process over CuSe.

When CuSe (or NiCuSe) thin films are exposed to visible light with energy equal to or exceeding the band-gap, it propels valence band electrons into the conduction band, generating positively charged holes (h^+^) in the valence band and negatively charged electrons (e^−^) in the conduction band of the thin film. This mechanism is predominantly governed by the intrinsic surface properties of the semiconductor. Holes and electrons migrate to the photocatalyst surface, initiating oxidation and reduction reactions with adsorbed species (h^+^_vb_ and e^−^_cb_), resulting in the formation of free radicals. In summary, the mechanism of this degradation process can be elucidated as follows: The vacancies formed in the valence band couple with hydroxide ions (OH^−^) derived from water ionization, resulting in the generation of hydroxyl free radicals (OH*). Simultaneously, the electrons in the conduction band associate with oxygen (O_2_), creating superoxide anion free radicals (O_2_*^−^). These O_2_*^−^ react with hydrogen ions (H^+^) to produce peroxide free radicals (HOO*). Subsequently, when exposed to ultraviolet light, the HOO* transform into OH*. Ultimately, these OH* lead to the degradation of CR dye, converting it into water or carbon dioxide^67,68^. Upon coupling with the electrons, the oxygen molecules in the solution creates superoxide anion radicals (O_2_*^−^). Meanwhile, with the aid of holes, water molecules undergo oxidation, producing hydroxyl radicals (OH*) which is crucial for the degradation of organic dye molecules. The efficiency of the photocatalytic reaction is enhanced by the separation of photoexcited carriers through charge transfer. The process can be found in detail in references^69,70^. Figure 9a and c depict the relationship between the absorbance of CR in an as-deposited CuSe and Ni-doped CuSe thin films after 1 and 2 h of irradiation, respectively. The observed reduction in absorbance over time is associated with the decreasing concentration of CR.

Figure 9a and b illustrate that CR undergoes degradation in the presence of CuSe under irradiation, and as the irradiation time increases, its destruction intensifies. Consequently, the absorption percentage decreases. This percentage is equivalent to 51% after 1 h of irradiation and 74% after 2 h. This phenomenon occurs for the Ni-doped sample with the presence of second band-gap, facilitating better light absorption, easier oxidation and reduction processes ^71^. Therefore, as observed in Fig. 9c and d, a degradation percentage of approximately 20% is seen after 1 h, and it reaches 86.5% after 2 h. It seems that the effectiveness of NiSe NiCuSe in the photocatalytic process requires more time. That is why after 1 h, a lower degradation percentage is observed in the doped state. However, after 2 h, the doped sample exhibits a more degradation, indicating a more significant impact. This observation aligns with the ratio of absorbance spectrum changes after 1 h and 2 h for both doped and pure CuSe, indicating that over time, the presence of Ni enhances the photocatalytic properties in CuSe.

## Conclusion

In this study, two metal ions (Ni, and Cu) were utilized to form metal selenide, resulting in the creation of a Ni-doped CuSe thin film. The chemical solution deposition method was employed, varying the amounts of Ni (0.01, 0.02, and 0.03 mol) and deposition times (3, 4, 5, and 6 h). The results showed that adding Ni to CuSe effectively generated second band-gaps (second optical transitions) and improved the structural quality. This subject is not easily observable and investigable using the Tauc method. Structural analysis confirmed the successful incorporation of Ni into the CuSe structure, enhancing crystallinity. Morphological findings indicated that Ni enhanced nucleation and cluster formation, preventing layer discontinuity with better substrate coverage. Additionally, Ni significantly reduced pinholes and created particles of different sizes. Optical investigations revealed that Ni induced the formation of a second transition (band-gap) at lower energy, contributing to effective photocatalytic performance. Moreover, prolonged deposition time and increased Ni molarity resulted in a reduction in the second band-gap. Furthermore, the Derivation Ineffective Thickness Method (DITM) was employed to overcome challenges in calculating the optical band-gap compared to the Tauc model (inability to display the second transition). The DITM method provided more accurate band-gap values without requiring knowledge of layer thickness or assumptions about the type of transition. Finally, the direct band-gap of Ni-doped CuSe was confirmed, demonstrating the positive impact of adding Ni in the photocatalytic application of CuSe.

## Data Availability

This manuscript presents original analyses, data and findings derived from independent research and are not sourced from external repositories. The dataset is exclusively accessible to the authors and is not publicly available. All necessary data and analyses are comprehensively presented in the manuscript, eliminating the need for additional materials.
